# Clinical validation of free breathing respiratory triggered retrospectively cardiac gated cine balanced steady-state free precession cardiovascular magnetic resonance in sedated children

**DOI:** 10.1186/s12968-014-0101-1

**Published:** 2015-01-14

**Authors:** Rajesh Krishnamurthy, Amol Pednekar, Lamya A Atweh, Esben Vogelius, Zili David Chu, Wei Zhang, Shiraz Maskatia, Prakash Masand, Shaine A Morris, Ramkumar Krishnamurthy, Raja Muthupillai

**Affiliations:** EB Singleton Department of Pediatric Radiology, Texas Children’s Hospital, Baylor College of Medicine, 6701 Fannin St, Suite 1280, Houston, TX 77030 USA; Philips Healthcare, MR Clinical Science Group, NA 595, Miner Road, Highland Heights, OH 44143 USA; Department of Radiology, St. Luke’s Episcopal Hospital and Texas Heart Institute, 6720 Bertner Ave, Houston, TX 77030 USA

**Keywords:** Respiratory triggered cine SSFP, Congenital heart disease, Cardiovacualr magnetic resonance, Sequence, Pediatrics

## Abstract

**Background:**

Cine balanced steady-state free precession (SSFP), the preferred sequence for ventricular function, demands uninterrupted radio frequency (RF) excitation to maintain the steady-state during suspended respiration. This is difficult to accomplish in sedated children. In this work, we validate a respiratory triggered (RT) SSFP sequence that drives the magnetization to steady-state before commencing retrospectively cardiac gated cine acquisition in a sedated pediatric population.

**Methods:**

This prospective study was performed on 20 sedated children with congenital heart disease (8.6 ± 4 yrs). Identical imaging parameters were used for multiple number of signal averages (MN) and RT cine SSFP sequences covering both the ventricles in short-axis (SA) orientation. Image quality assessment and quantitative volumetric analysis was performed on the datasets by two blinded observers. One-sided Wilcoxon signed rank test and Box plot analysis were performed to compare the clinical scores. Bland-Altman (BA) analysis was performed on LV and RV volumes.

**Results:**

Scan duration for SA stack using RT-SSFP (3.9 ± 0.8 min) was slightly shorter than MN-SSFP (4.6 ± 0.9 min) acquisitions. The endocardial edge definition was significantly better for RT than MN, blood to myocardial contrast was better for RT than MN without reaching statistical significance, and inter slice alignment was comparable. BA analysis indicates that the variability of volumetric indices between RT and MN is comparable to inter and intra-observer variability reported in the literature.

**Conclusions:**

The free breathing RT-SSFP sequence allows diagnostic images in sedated children with significantly better edge definition when compared to MN-SSFP, without any penalty for total scan time.

## Background

Cine balanced steady state free precession (SSFP) is the cardiovascular magnetic resonance (CMR) technique of choice for assessing ventricular size (end-diastolic volume, end-systolic volume), global function (ejection fraction) and regional function (wall motion, wall thickening) due to its a) high intrinsic blood to muscle contrast that is preserved throughout the cardiac cycle, b) high signal-to-noise ratio, and c) balanced gradient structure in all directions, which provides desirable flow properties [1-7]. However, cine SSFP sequences are sensitive to perturbations of the steady state due to field inhomogeneity or bulk–motion (blood flow and myocardial motion) that can cause image artifacts due to initial oscillatory transient signal, and blood to myocardial contrast change during the approach to steady state. In addition, it requires a minimum repetition time (TR) between radio frequency (RF) pulses to reduce banding artifacts. Thus, cine SSFP sequence has conflicting constraints of uninterrupted excitations (single shot), specific absorption rate (SAR) limit affecting the minimum TR, and respiratory motion compensation [4,8]. In routine clinical practice, cine SSFP acquisitions are therefore performed during suspended respiration (breath-holding) using a single continuous shot of excitations, and the spatial and temporal resolution are adapted to fit within a single breath-hold session. Conventional cine SSFP techniques typically require 10 to 12 breath holds of 8 to 10 seconds each to cover the entire LV with a temporal resolution of 30 to 45 msec. These successive breath holds are difficult to accomplish in pediatric patients. Even older children may have difficulty holding their breath consistently at the same respiratory level. Younger children who cannot cooperate by lying still for the duration of the study typically undergo general endotracheal anesthesia (GETA) to enable breath-held imaging. The challenges in children, even when intubated, are compounded by the need for higher spatial resolution (small structures) and higher temporal resolution (rapid heart rates) [9]. There is a growing preference in children’s hospitals towards the use of intravenous sedation rather than GETA for CMR since it is considered to be safe, less invasive and more physiologic, but requires adaptation of the CMR sequences for free-breathing acquisition. A commonly used strategy in this setting is to average the signal over multiple bSSFP acquisitions (multiple NSA or MN) during free-breathing to minimize the effect of respiratory motion [4]. This approach poses special challenges. Firstly, the respiratory bulk motion makes the tissues being imaged in short-axis orientation to fall in and out of steady state, introducing a potential source of artifacts if the diaphragmatic excursion is significant. Secondly, the relatively large flip angles and short TR in SSFP imaging make cumulative RF dose a source of concern in multi-phase, multi-slice, multi-NSA acquisitions. Thirdly, the through plane motion over several cardiac cycles causes blurring of the endocardial boundary, affecting volumetric measurements. The alternative to multi-NSA acquisition is real time dynamic imaging without cardiac or respiratory synchronization. While this is useful, both these imaging strategies often make tradeoffs in temporal resolution and/or spatial resolution to keep the RF dose within prescribed limits. In addition, while real time imaging makes it possible for qualitative assessment of LV function, quantitative assessment is difficult.

We propose a free breathing respiratory triggered multi-shot cardiac cine SSFP technique (RT-SSFP) with a drive to steady state before each expiration, coupled with arrhythmia rejection and retrospective cardiac gating. The purpose of this study is to compare the RT-SSFP sequence to the multiple number of signal averages (MN-SSFP) technique that is currently used in routine clinical practice for assessment of ventricular function in freely breathing sedated pediatric patients.

## Methods

### Study population

The studies were performed prospectively on a group of 20 consecutive children with congenital heart disease undergoing clinically indicated CMR. The group included 14 males and 6 females, with age 8.6 ± 4 (range 2–17) years, heart rate 94 ± 17 bpm (range 75–139), and respiration rate: 20.6 ± 5.6 rpm (range 13–36). The indications for the studies were as follows: 7 tetralogy of Fallot repair, 4 aortic coarctation repair, 3 pulmonary stenosis repair, and 1 each with constrictive pericarditis, myocarditis, suspected Kawasaki disease, bicuspid aortic valve with aortic dilation, double outlet right ventricle and D-transposition of great arteries status post arterial switch, and William syndrome with supravalvular aortic stenosis and branch pulmonary artery stenosis. The study was approved by the Institutional Ethics Committee and complied with the Health Insurance Portability and Accountability Act of 1996. All subjects gave written informed consent before being enrolled in the study. 18 patients received intravenous sedation, while 2 patients were able to cooperate with lying still and breathing freely without sedation.

### Image acquisition

**Figure 1:**
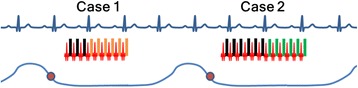
**Respiratory triggered cine bSSFP acquisition schematic.** RF excitations starts at user-defined time delay after user-specified respiratory expiration trigger. Case 1: R wave occurs at time longer than user-prescribed time to steady state after the beginning of RF excitations, DAQ is turned on and data is accepted until following R wave occurs and R-R interval is within prescribed arrhythmia rejection window. Case 2: R wave occurs in less than user-prescribed time to steady state, the DAQ is turned on only after the subsequent R wave and data is accepted as in Case 1. Phase encoding steps for the shot are changed after successful acceptance of the previous shot. Red dot – expiration trigger, RF- radio frequency, DAQ – data acquisition, black rectangle – DAQ off, orange/green rectangle – DAQ ON.

All imaging was performed on a commercial 1.5 T MR scanner (Achieva, Philips Healthcare, Best, The Netherlands) using a 5-channel phased-array surface coil and vector-cardiographic (VCG) gating. The CMR protocol for all 20 patients involved acquisition of scout images of the thoracic cavity along the 3 orthogonal planes that were used as a guide for the following series of retrospectively VCG-gated cine bSSFP images acquired during normal respiration. Identical image acquisition parameters were used for 2D free-breathing MN and RT cine SSFP sequences covering both the ventricles [4] with a series of 10 to 14 contiguous slices in short-axis (SA) orientation. The acquisition parameters were: TR/TE/flip angle: 3 ms/1.5 ms/60°; acquired voxel size: (1.5-1.9) × (1.5-2.1) × (7–8) mm^3^; Sensitivity Encoding (SENSE) acceleration factor: 2; temporal resolution: 30–45 ms per cardiac phase. The absolute total data acquisition time excluding the arrhythmia rejection and gating was 8–10 RR intervals per slice depending on patient size and heart rate. The MN scan used an average of four signal acquisitions with real-time arrhythmia rejection and retrospective reconstruction. In order to perform RT scan, the vendor-provided stock cine SSFP sequence was modified to include respiratory triggering with the ability to have a user-prescribed trigger point and trigger delay, and a user-prescribed minimum duration for start-up excitations (to drive the magnetization to steady state) before commencing the retrospectively cardiac gated acquisition. In 17 patients with respiratory rate below 30 per minute the respiratory trigger point was set at the beginning of the expiration with a trigger delay of 100–200 ms to ensure that drive to steady state also happened in the quiescent respiratory period. In 3 patients with respiratory rate of greater than 30 per minute, an inspiratory trigger was used with 500–700 ms trigger delay, so that image acquisition commenced during early expiration. The start-up RF excitations to drive the magnetization to steady state was set to 300–400 ms (time to attain steady state by myocardial tissue at 1.5 T), before looking for an R wave from the VCG signal [5]. The data acquisition commenced at the occurrence of the R wave, and ended at the occurrence of the following R wave, and was processed by the real-time arrhythmia rejection algorithm (Figure 1). After completion of the data acquisition, cardiac phase data was processed by the retrospective cardiac gating algorithm in the reconstruction phase. Both MN and RT scans used Philips’ commercial real-time arrhythmia rejection with acceptance window of ±10-15% of user-defined heart rate and retrospective cardiac gating. In this scheme, data for specific phase encoding steps is repeatedly acquired during a complete RR interval. Real-time arrhythmia rejection algorithm directs the system to reject and re-measure data of an RR-interval if the next R-peak is not within the RR-window (RR interval either too short or too long). This process is performed until all phase encoding steps are recorded. Retrospective reconstruction algorithm matches the VCG and the phase encoding timings and performs non-linear time scaling for the data with ±10-15% RR variation, thus providing multiple cardiac phases with full RR coverage. The total scan duration to acquire images from the entire left ventricle using both RT-SSFP and MN-SSFP was noted.

### Image analysis

All qualitative and quantitative image analysis was performed by two CMR experts with 13 and 3 years of experience respectively, who were blinded to the image acquisition technique used. Qualitative comparison between the two techniques was performed using the clinical scores based on three image quality parameters: a) blood-myocardial contrast (BMC), b) edge definition (EDef), and c) interslice alignment (ISA) visualized on a long-axis projection of the short-axis stack. Each parameter was graded on a scale from 1 to 3. The clinical scoring system was as follows: BMC: 1- excellent (distinctly hypo-intense myocardium w.r.t. blood pool), 2-good (noticeable), 3-adequate (discernible); EDef: 1-excellent (sharp), 2-good (definable), 3-poor (blurry); ISA: 1-All slices aligned, 2- <2 slices misaligned, 3- >2 slices misaligned. Volumetric post-processing was performed on a dedicated workstation (ViewForum; Philips Healthcare, Best, The Netherlands). LV and RV functions were evaluated quantitatively by manually drawing the endocardial contours at end-diastole (the phase with the largest blood-pool cavity) and end-systole (the phase with the smallest blood-pool cavity). Quantitative volumetric analysis performed included right and left ventricular end diastolic volume (EDV), and end systolic volume (ESV). Right and left ventricular ejection fractions (EF) were calculated from the ESV and EDV.

### Statistical analysis

Wilcoxon signed rank test was performed to compare the RT-SSFP clinical scores against MN-SSFP. Bland-Altman analysis was performed for EDV, ESV, and EF values of LV and RV to assess the degree of agreement between the two acquisition techniques.

## Results

**Figure 2:**
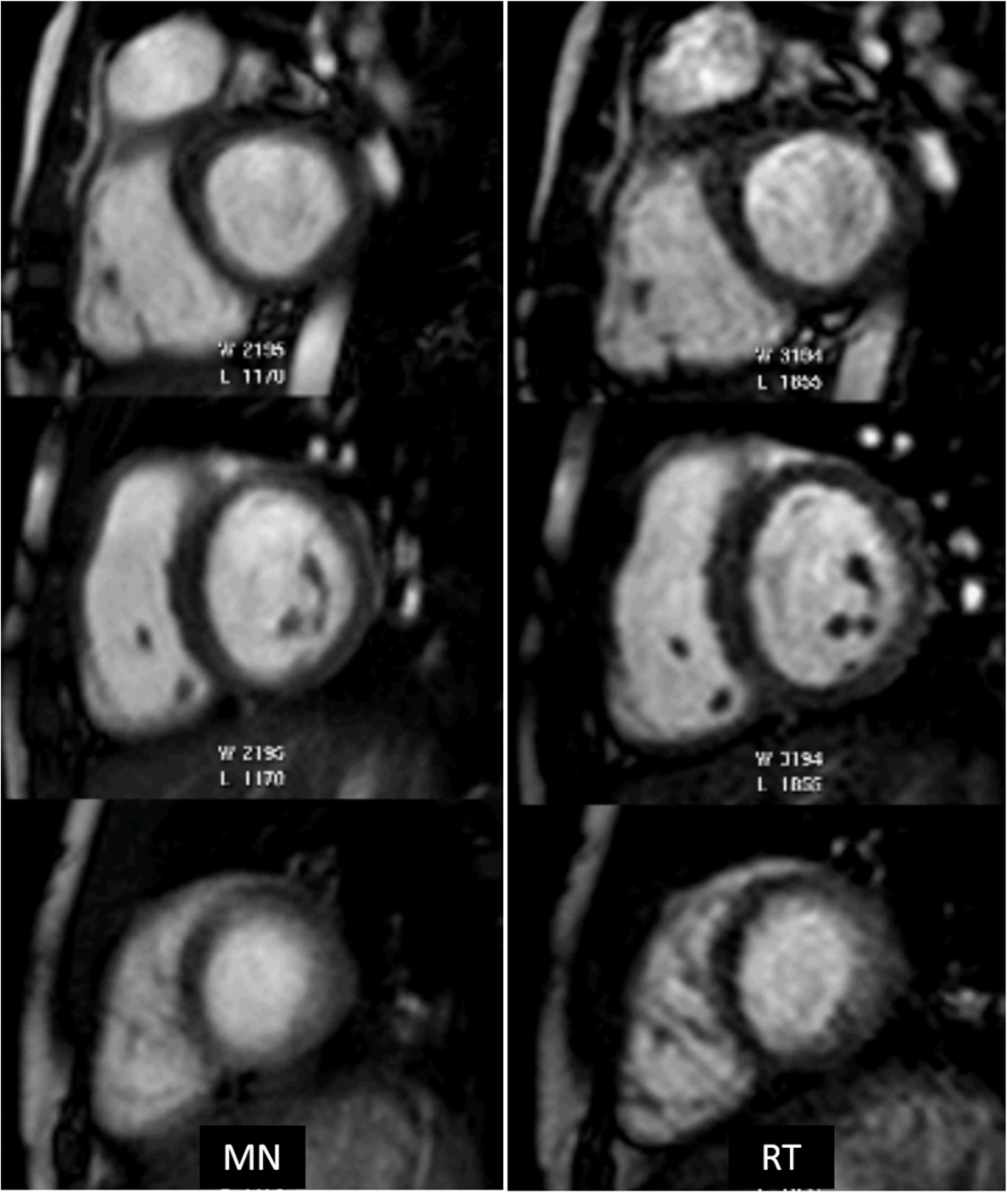
**Representative cardiac cine SSFP images with multiple signal averages (MN) and respiratory triggered (RT) acquisition techniques in an unsedated free-breathing 14 year old female with Down syndrome and status post Tetralogy of Fallot repair.** Three representative short axis slices from the base (top row), mid chamber (middle row) and apex (bottom row) are presented. The MN images on the left demonstrate subtle blurring of the myocardium and trabeculae, best visualized on the apical slice, while the RT images on the right show significant improvement in edge definition.

**Figure 3:**
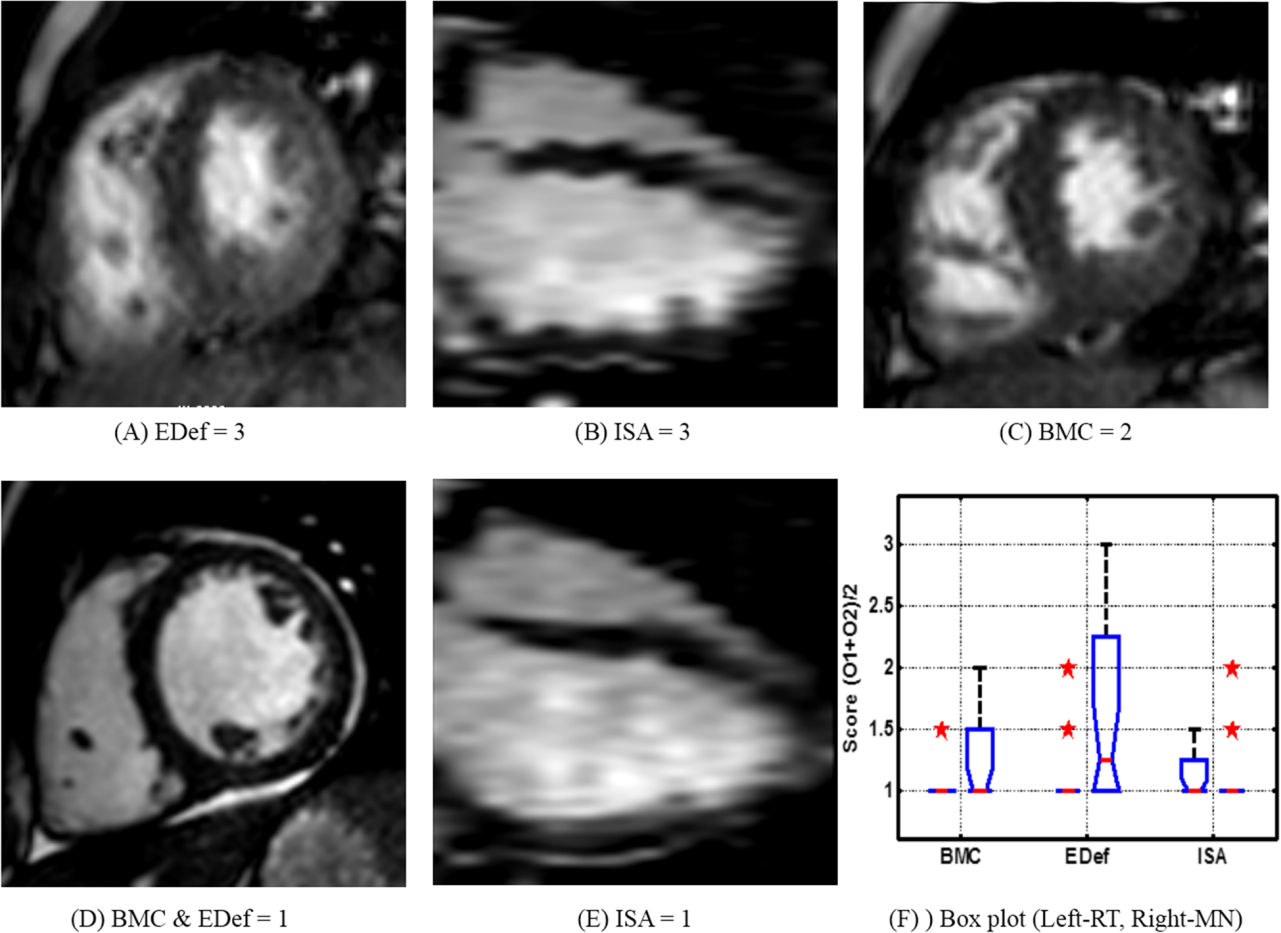
**(A-E) Representative images for the clinical scores for different categories and the spread and distribution of the score.** The clinical scoring system was as follows: BMC: 1- excellent (distinctly hypo-intense myocardium w.r.t. blood pool), 2-good (noticeable), 3-adequate (discernible); EDef: 1-excellent (sharp), 2-good (definable), 3-poor (blurry); ISA: 1-All slices aligned, 2- <2 slices misaligned, 3- >2 slices misaligned. BMC score reflects quality of the steady state, and EDef score indicates through plane motion blurring. ISA was determined by visualizing the SA stack as a volume. **(F)** Box plot for clinical scores depicts the spread and distribution of the clinical scores where non-overlapping notches indicate that the medians of the two groups differ at the 5% significance level. O1 – Observer 1; O2 – Observer 2.

Both MN and RT sequences were acquired successfully on all the patients. Total scan duration for SA stack using RT-SSFP (3.9 ± 0.8 min) was slightly shorter than MN-SSFP (4.6 ± 0.9 min) acquisitions (p-value = 0.01). Representative images acquired from a subject using MN and RT SSFP acquisition techniques are depicted in Figure 2. A total of 254 MN and 254 RT slices were clinically scored and manually contoured by two blinded observers. Figure 3 A-E demonstrates the qualitative difference between the clinical scores. The clinical scores for RT-SSFP were excellent in all three categories (BMC = 1.05 ± 0.22, EDef = 1.10 ± 0.30, and ISA = 1.15 ± 0.37). The scores for MN-SSFP were excellent for BMC (1.25 ± 0.44) and ISA (1.20 ± 0.41), and poorer for EDef (1.65 ± 0.75). One-sided Wilcoxon signed rank test indicated that while ISA (p = 1) and BMC (p = 0.22) scores were comparable, the EDef (p = 0.02) scores were significantly better for RT-SSFP than MN-SSFP (Table 1). The spread and distribution of the clinical scores is depicted in the notched box plot (Figure 3F) where non-overlapping notches indicate that the medians of the two groups differ at the 5% significance level. Overall, all the data sets were scored to be of diagnostic quality, with the clinical scores for EDef indicating significant improvement of edge definition in RT-SSFP compared to MN-SSFP. Total normalized score (with equal weights to each clinical score category) was significantly better for RT compared to MN (1.08 ± 0.15 compared to 1.31 ± 0.38 with p = 0.02; Table 1). Bland Altman analysis indicating the variability in LV and RV volumetric indices between RT and MN acquisitions is presented in Table 2, and the result is comparable to inter-observer variability reported in the literature for breath-held SSFP [7].

**Table 1:** **Statistical analysis of clinical scores using average of two observer scores ((O1 + O2)/2)**

**Table 1: RT vs. MN (n = 20)**								
	**BMC**		**EDef**		**ISA**		**Total normalized score**	
	**RT**	**MN**	**RT**	**MN**	**RT**	**MN**	**RT**	**MN**
Score = 1	19 (95%)	15 (75%)	18 (90%)	10 (50%)	17 (85%)	16 (80%)	14	10
Score > 1	1 (5%)	5 (25%)	2 (10%)	10* (50%)	3 (15%)	4 (20%)	6	10
Mean ± std	1.05 ± 0.22	1.25 ± 0.44	1.10 ± 0.30	1.65 ± 0.75	1.15 ± 0.37	1.20 ± 0.41	1.08 ± 0.15	1.31 ± 0.38
Median	1.00	1.00	1.00	1.50	1.00	1.00	1.00	1.08
P-value	0.22		0.02		1.00		0.02	

*4 of the 10 scores were graded >2 (poor).

**Table 2:** **Bland Altman analysis indicating the variability in LV and RV volumetric indices between RT and MN acquisitions**

**Table 2: BA Analysis RT vs. MN (n = 20)**						
	**LV**			**RV**		
	**EDV ml**	**ESV ml**	**EF%**	**EDV ml**	**ESV ml**	**EF%**
Bias	4.68	2.74	1.62	5.87	3.90	1.40
Limit of Agreement	9.51	4.63	5.81	12.27	7.24	9.43

## Discussion

In our study, we show that cine RT-SSFP imaging offers diagnostic image quality with better clinical score in comparison to the standard MN-SSFP imaging in sedated children. In all patients, steady state was attained successfully with optimal flip angle required for maximum blood to myocardial contrast at 1.5 T. The clinical scores for endocardial definition in RT were significantly better than MN, since data acquisition occurs during expiration and avoids the motion blurring seen in MN acquisitions. We also noted a trend towards improved myocardial-blood pool contrast for RT versus MN, but this did not reach statistical significance. The combination of prospective respiratory triggering and retrospective cardiac gating with drive to steady state in each respiratory cycle addresses both the spatio-temporal resolution and RF dose limitations of acquisition during free breathing. Total scan durations for the two techniques are comparable, with an added advantage of reduction in RF duty cycle with RT compared to MN. The reduction in effective SAR in RT can easily be used to improve spatio-temporal resolution compared to MN acquisition and help acquire 3D images that can enable multi-planar reformatting. The ventricular volumetric measurements were in close agreement. The inconsistent respiration levels between RT and MN may lead to discrepancy in the estimation of basal volume and can explain the variability in EDV and ESV using two different acquisitions, especially for the RV. The enhanced edge definition may be beneficial for the automatic LV segmentation and wall motion analysis algorithms.

In order to minimize motion artifacts, imaging in quiescent phases of respiration is preferred [2,9]. Therefore image acquisition is prescribed in the expiratory phase. This requires manual input on the part of the operator for choice of inspiratory versus expiratory trigger, time between the trigger and the beginning of start-up excitations (trigger delay), and the duration of start-up excitations (acquisition delay). In children with a respiratory rate of less than 30 per minute with heart rate above 80 bpm, there is enough time during expiration to drive the magnetization to steady state and complete acquisition prior to the onset of the next inspiration [9]. In patients with a respiratory rate of greater than 30 per minute, an inspiratory trigger is used, with a user-defined trigger delay prior to onset of start-up RF excitations to drive the magnetization to steady state, so that image acquisition is timed to commence at the start of expiration.

### Limitations

There are some limitations to this study. First, this study involved a small, but representative pediatric population, and will require validation in a larger cohort. Secondly, user input is needed to choose an inspiratory versus expiratory trigger, and to adjust the duration of the trigger delay and startup RF excitations based on the respiratory rate. Thirdly, in the setting of high respiratory rates and slow heart rates, the acquisition can spill over into the next inspiratory cycle, resulting in motion blurring. The next iteration of the sequence will have the ability to automatically select the respiratory trigger based on the monitored respiratory rate, and to retrospectively bin the data based on the cardiac and respiratory cycle, and reject data acquired during inspiration.

## Conclusion

It is feasible to use a cine bSSFP sequence combined with prospective respiratory triggering and retrospective cardiac gating to assess LV function in free breathing patients. Such a sequence can generate images with high myocardium to blood contrast, temporal resolution, and signal-to-noise ratio intrinsic to balanced SSFP sequences. RT acquisition is superior to MN in terms of image quality, primarily related to improved edge definition, is slightly shorter than MN imaging for total scan duration, and is comparable to MN for quantifying LV and RV volumes and function in sedated pediatric patients.

## Abbreviations

SSFP: Steady state free precession
RT: Respiratory triggered
NSA: Number of signal averages
MN: Multiple NSA
Edef: Edge definition
BMC: Blood myocardial contrast
ISA: Inter-slice alignment
VCG: Vector electrocardiographic gating
RF: Radio frequency
LV: Left ventricle
RV: Right ventricle
SA: Short axis
CMR: Cardiovascular magnetic resonance
